# Supplementary material for: Patterns of satellite tagged hen harrier disappearances suggest widespread illegal killing on British grouse moors
Source: Nat Commun. 2019 Mar 19;10:1094. doi: 10.1038/s41467-019-09044-w (PMC6424969; doi:10.1038/s41467-019-09044-w)
Supplement: Supplementary file 1 — Supplementary Information [file 41467_2019_9044_MOESM1_ESM.pdf]

Supplementary Information

Patterns of satellite tagged hen harrier disappearances suggest widespread illegal killing on British grouse moors

Murgatroyd et al.

Supplementary Table 1: Summary of all hen harrier tracking data used in analyses.

Bird identity	Fate	*n* fixes	*n* days	*n* terminal fixes
161143	A	326	94	na
162150	A	383	90	na
162149Mo	A	394	97	na
162148	A	1499	448	na
161962	A	1703	450	na
117314	A	2955	1535	na
117313	A	3008	1552	na
137372Na	N	13	13	11
137372Jo	N	84	34	33
74926*	N	117	32	42
94588	N	342	241	5
94592	N	402	175	6
58946*	I	88	30	14
162149Ro	I	373	98	19
58946An*	I	601	270	15
74843	I	845	355	26
94590	SIM	8	3	8
73589	SIM	17	90	3
73582	SIM	19	108	6
73588	SIM	28	93	6
73586	SIM	47	91	4
90690	SIM	54	34	14
147107	SIM	70	24	25
73591	SIM	73	103	13
73590	SIM	79	110	8
58870	SIM	85	40	23
94589	SIM	92	49	19
94591	SIM	121	56	26
73584	SIM	136	333	21
90688	SIM	150	79	22
90687	SIM	165	126	10
74843Ja	SIM	174	45	41
137369	SIM	195	67	28
94588Ba	SIM	205	57	32
147109	SIM	239	74	29
73585	SIM	245	112	2
33335	SIM	255	397	8
74931*	SIM	273	71	39
90689	SIM	280	103	21
33325	SIM	289	359	16
90691	SIM	291	239	14
33334	SIM	307	750	1
58867	SIM	309	160	11

137859	SIM	323	84	46
58943	SIM	339	63	46
58945	SIM	361	149	8
161961	SIM	388	93	37
137372Jo2	SIM	433	141	22
74832	SIM	438	106	36
117316	SIM	440	88	43
74842	SIM	451	138	8
58941	SIM	471	140	2
161147	SIM	485	155	9
147108	SIM	732	250	28
33328	TF	6	10	na
58872	TF	915	522	na
73587	TF	84	402	na
117315	TF	1242	374	na

Fate abbreviations: A: Alive and still transmitting, TF: Transmitter malfunctioned (i.e. the tag ceased transmitting but the bird was confirmed to be alive visually) or tag failure likely due to diagnostic plots, SIM: Suspected Illegal Mortality, I: Bird dead, confirmed to have been illegally killed or tag dropped under suspicious circumstances (i.e. the tag was recovered with no harness damage and the bird had been removed from the harness), N: Bird recovered and cause of death established to be natural. All tags were set to duty cycle 8 hours on and 48 hours off, except * which had 10 hours on 60 hours off. *n* fixes; total number of fixes (Location class 3, 2, 1, 0), *n* days; total number of days since tagging until last known fix (or cut-off at 5th Oct 2017 for alive birds). *n* terminal fixes; total number of fixes in the last week of tracking.

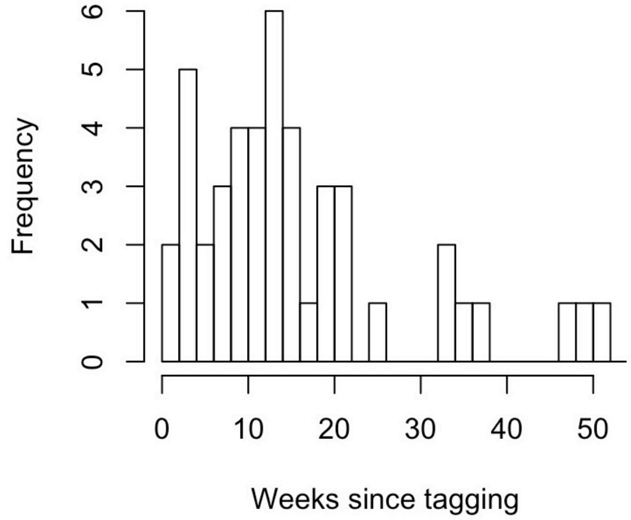

Supplementary Figure 1: The frequency of hen harrier deaths or disappearances during the first year of life.

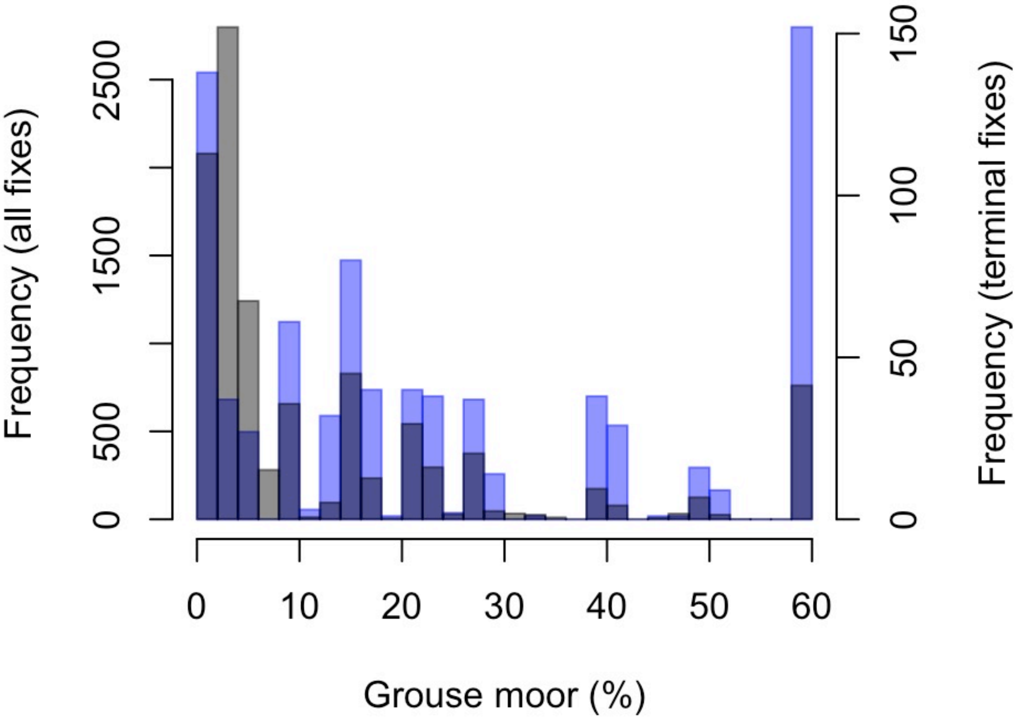

Supplementary Figure 2: The frequency of all (i.e. live and terminal fixes) hen harrier locational fixes on managed grouse moors in the UK (grey bars) and the same frequency for fixes in the terminal week of life only (z-axis, blue bars). These data are from birds that were suspected or known to have been illegally killed.

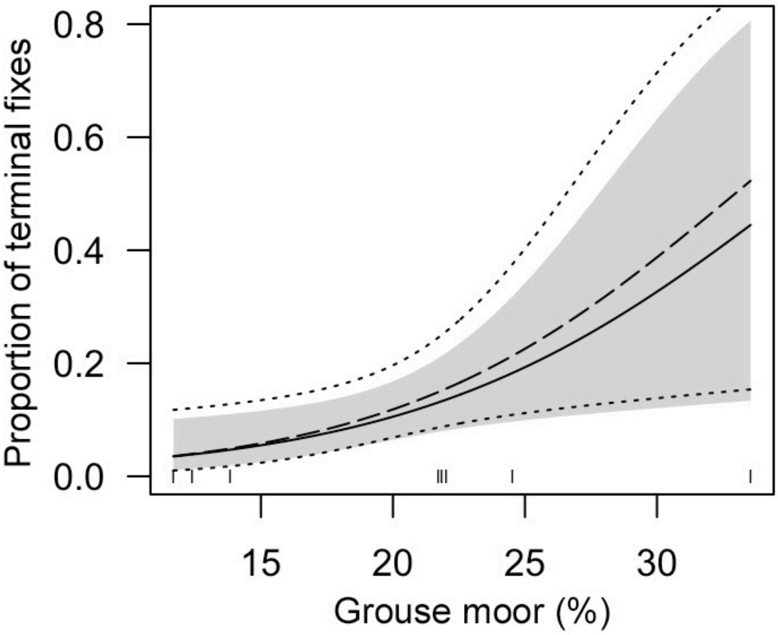

Supplementary Figure 3: Relationship between probability of last week and percentage grouse moor habitat in protected areas in the UK. *dashed lines are for harriers suspected or known to have been illegally killed, dotted are their CIs. Solid line and grey CIs are for all tracked harriers.

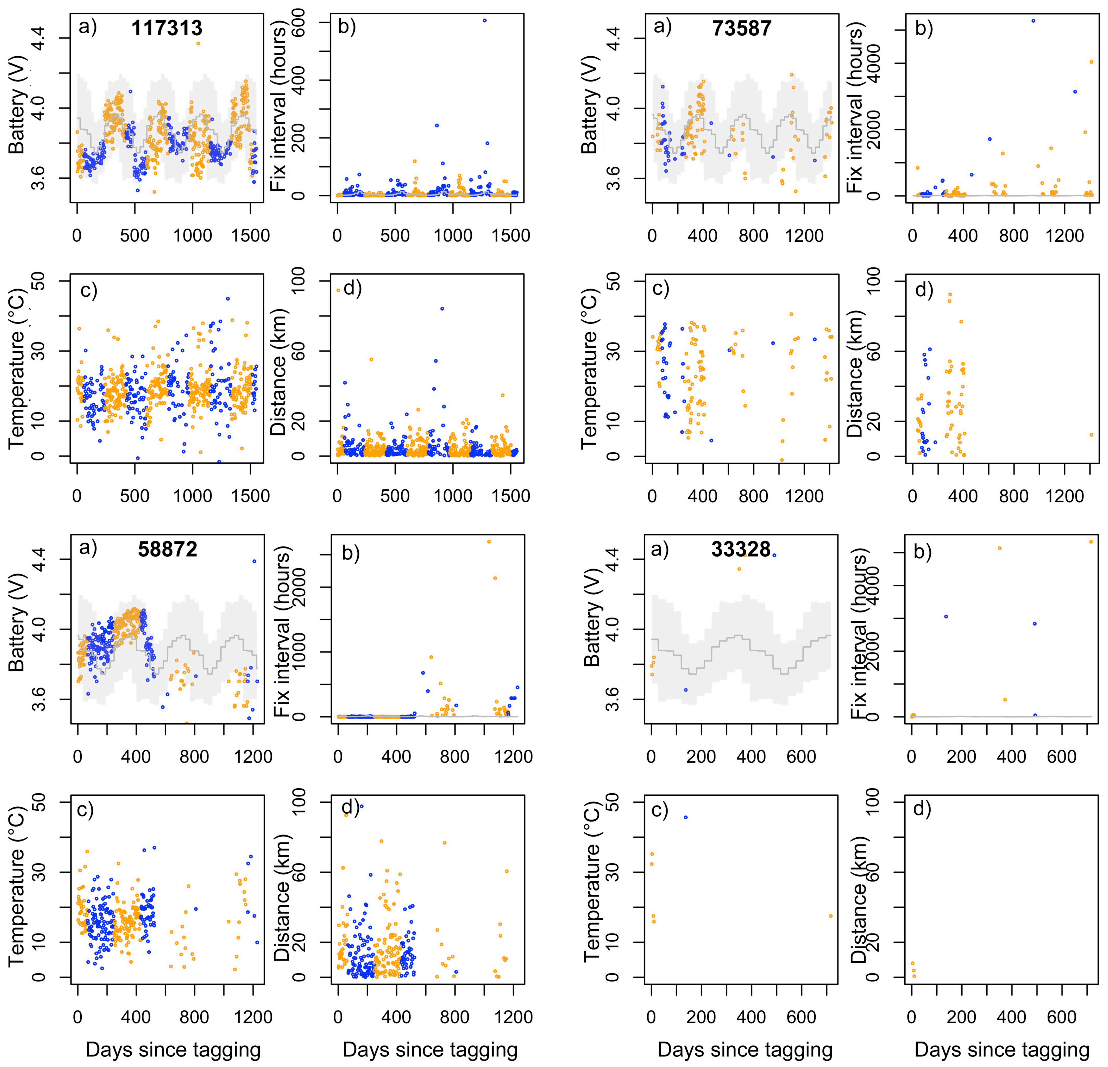

Supplementary Figure 4: Diagnostic plot examples for four tags: (a) Mean daily battery voltage. This indicated a voltage level outside of the 'normal' range for that time of year. Voltage follows a sinusoidal pattern through the seasons, thus to understand the 'normal' range we also plotted the average (± standard deviation) voltage for all transmitters. These were trimmed by 30 days at the start and end of tracking to remove any potentially inaccurate data; e.g. failing battery at end of tracking period, for each month of the year (regardless of year); (b) Mean daily fix interval. Frequent high (>100 hours) values or long delays not corresponding with low voltage during winter months were indications of tag failure; (c) Mean temperature. Sudden unexpected fluctuation in the temperature sensor can be indicative of a possible imminent tag failure (Whitfield and Fielding 2017); (d) Mean daily distance moved. Highly fluctuating distances moved can be indicative of antenna damage and frequent failure to secure accurate fixes. Point colours broadly separate summer (orange) and winter (blue) months. 117313 is alive and still tracking, this diagnostic plot is typical of a healthy tag with annual cyclic fluctuations in battery and temperature and low fix interval and distance travelled. These patterns are also present on tags which were classified an "stopped no malfunction"; whereby usual diagnostics were followed by sudden cessation of tracking. 75387 tracker failed and this bird was subsequently photographed alive. Fix intervals are sporadic and distance travelled is so large it become outside the y-axis limit indicating poor fix accuracy. 58872 and 33328 are the only trackers classified as failed due solely to diagnostic plots.
