# Supplementary material for: Patterns of satellite tagged hen harrier disappearances suggest widespread illegal killing on British grouse moors
Source: Nat Commun. 2019 Mar 19;10:1094. doi: 10.1038/s41467-019-09044-w (PMC6424969; doi:10.1038/s41467-019-09044-w)
Supplement: Supplementary file 3 — Reporting Summary [file 41467_2019_9044_MOESM3_ESM.pdf]

natureresearch

Corresponding author(s): Megan Murgatroyd

Last updated by author(s): Jan 25, 2019

# Reporting Summary

Nature Research wishes to improve the reproducibility of the work that we publish. This form provides structure for consistency and transparency in reporting. For further information on Nature Research policies, see Authors & Referees and the Editorial Policy Checklist.

## Statistics

For all statistical analyses, confirm that the following items are present in the figure legend, table legend, main text, or Methods section.

| n/a | Confirmed | |
|---|---|---|
| ☐ | ☒ | The exact sample size (*n*) for each experimental group/condition, given as a discrete number and unit of measurement |
| ☒ | ☐ | A statement on whether measurements were taken from distinct samples or whether the same sample was measured repeatedly |
| ☐ | ☒ | The statistical test(s) used AND whether they are one- or two-sided<br>*Only common tests should be described solely by name; describe more complex techniques in the Methods section.* |
| ☐ | ☒ | A description of all covariates tested |
| ☐ | ☒ | A description of any assumptions or corrections, such as tests of normality and adjustment for multiple comparisons |
| ☐ | ☒ | A full description of the statistical parameters including central tendency (e.g. means) or other basic estimates (e.g. regression coefficient) AND variation (e.g. standard deviation) or associated estimates of uncertainty (e.g. confidence intervals) |
| ☐ | ☒ | For null hypothesis testing, the test statistic (e.g. *F*, *t*, *r*) with confidence intervals, effect sizes, degrees of freedom and *P* value noted<br>*Give P values as exact values whenever suitable.* |
| ☒ | ☐ | For Bayesian analysis, information on the choice of priors and Markov chain Monte Carlo settings |
| ☒ | ☐ | For hierarchical and complex designs, identification of the appropriate level for tests and full reporting of outcomes |
| ☐ | ☒ | Estimates of effect sizes (e.g. Cohen's *d*, Pearson's *r*), indicating how they were calculated |

*Our web collection on statistics for biologists contains articles on many of the points above.*

## Software and code

Policy information about availability of computer code

| | |
|---|---|
| Data collection | All data was collected using satellite transmitters manufactured by Microwave telemetry Inc, Columbia, Maryland, USA |
| Data analysis | All analysis was performed using publicly available packages within R version 3.3.0 |

For manuscripts utilizing custom algorithms or software that are central to the research but not yet described in published literature, software must be made available to editors/reviewers. We strongly encourage code deposition in a community repository (e.g. GitHub). See the Nature Research guidelines for submitting code & software for further information.

## Data

Policy information about availability of data

All manuscripts must include a data availability statement. This statement should provide the following information, where applicable:

- Accession codes, unique identifiers, or web links for publicly available datasets
- A list of figures that have associated raw data
- A description of any restrictions on data availability

The complete datasets analysed in this study are not publicly available due to the sensitivity of the locational data, but are available from the corresponding author on reasonable request and with permission of Natural England. The source data for the Figs 1 and 2 have been provided as a Source Data file.

# Field-specific reporting

Please select the one below that is the best fit for your research. If you are not sure, read the appropriate sections before making your selection.

☒ Life sciences ☐ Behavioural & social sciences ☐ Ecological, evolutionary & environmental sciences



October 2018

# Life sciences study design

All studies must disclose on these points even when the disclosure is negative.

| | |
|---|---|
| Sample size | Sample sizes are given as the number of individual fixes and the number of bird-weeks which tracking data was collected for. All sample sizes are clearly summarised in Table 2 in the manuscript. |
| Data exclusions | The tracking data collected included outliers due to less accurate fix quality. Thus we removed any duplicate fixes, retaining only those fixes with the best quality LC (3, 2, 1, 0) and highest battery voltage |
| Replication | NA |
| Randomization | Experimental groups were not allocated |
| Blinding | Data collection and analyses were performed by different authors. No blinding was nessecary |

# Reporting for specific materials, systems and methods

We require information from authors about some types of materials, experimental systems and methods used in many studies. Here, indicate whether each material, system or method listed is relevant to your study. If you are not sure if a list item applies to your research, read the appropriate section before selecting a response.

## Materials & experimental systems

| n/a | Involved in the study | |
|---|---|---|
| ☒ | ☐ | Antibodies |
| ☒ | ☐ | Eukaryotic cell lines |
| ☒ | ☐ | Palaeontology |
| ☐ | ☒ | Animals and other organisms |
| ☒ | ☐ | Human research participants |
| ☒ | ☐ | Clinical data |

## Methods

| n/a | Involved in the study | |
|---|---|---|
| ☒ | ☐ | ChIP-seq |
| ☒ | ☐ | Flow cytometry |
| ☒ | ☐ | MRI-based neuroimaging |

## Animals and other organisms

Policy information about studies involving animals; ARRIVE guidelines recommended for reporting animal research

| | |
|---|---|
| Laboratory animals | NA |
| Wild animals | Wild hen harriers were fitted with tracking devices prior to fledging from the nest. This was done by a trained professional (coauther SGM). No birds were removed from the wild for this study. |
| Field-collected samples | NA |
| Ethics oversight | The hen harrier is listed on Schedule 1 of the Wildlife and Countryside Act 1981 (as amended) and all potentially disturbing work affecting breeding birds, and / or their dependent young, was carried out under licence by experienced individuals. The use of harness-mounted radio and satellite tags was approved by the Special Methods Technical Panel of the British Trust for Ornithology's Ringing Committee. Tracking work, using harness-mounted transmitters, was also approved by Natural England's Executive and Non-executive Board. |

Note that full information on the approval of the study protocol must also be provided in the manuscript.
